# Is there a valid ethical objection to the clinical use of *in vitro*-derived gametes?

**DOI:** 10.1530/RAF-21-0066

**Published:** 2021-11-23

**Authors:** Kristian Galea

**Affiliations:** 1 Gonville & Caius College, Trinity Street, Cambridge, UK

**Keywords:** gametogenesis, assisted reproduction, stem cells, ethics

## Introduction

Recent developments in techniques that can determine cell fate have allowed the differentiation of mouse embryonic stem cells and induced pluripotent stem cells into germ cells. Research conducted in the past decade has also made significant advances towards meeting this end using human embryonic stem cells. The technology is known as* in vitro* gametogenesis (IVG) and has potential as an assisted reproductive technology (ART). However, before this is possible, the ethical issues surrounding these techniques for research and reproduction must be assessed.

IVG can be carried out by allowing induced pluripotent stem cells to differentiate in culture conditions that closely resemble those to which a germ cell is exposed* in vivo* – this is known as directed differentiation. Research that delineated key factors of human germ cell specification (Irie *et al.* 2015, Sasaki *et al.* 2015) made this possible and expedited the development of culture methods that adequately reconstitute* in vivo* conditions. For instance, the induction of oogonia-like cells from human primordial germ cell-like cells was accomplished* in vitro* by culturing them with mouse embryonic ovarian somatic cells (Yamashiro *et al.* 2018).

IVG has potential research applications that could facilitate developments in the mechanistic understanding of infertility, epigenetic reprogramming and meiotic recombination, to name a few. In the clinic, IVG could form the basis of an ART for those who lack functional gametes but desire children with whom they share genetic material as in natural conception. Herein lies the value of IVG; it offers a benefit that is not offered by any other approved ARTs.

Despite its potential, current attitudes towards the clinical use of IVG are negative. However, these arguments tend to focus upon the safety concerns associated with IVG in its current form rather than in-principle ethical objections against the potential treatment. I argue that there is no justifiable in-principle ethical objection to the use of IVG as an ART for those who cannot have offspring with whom they share genetic material by any other means. For this reason, I conclude that regulatory bodies such as the Human Fertilisation and Embryology Authority (HFEA) must seriously consider IVG as a potential future ART with regard to its ethical status rather than its current feasibility.

## Separating safety concerns and ethical objections

In a systematic review conducted by Hendriks *et al.* (2015), it was found that the most frequently cited concerns were regarding the safety of IVG. These worries stem from the understandable paucity of evidence to suggest that IVG, in its current form, would be safe for clinical use.

However, while concerns over risk and uncertainty are of utmost importance to the implementation of IVG as an ART, they do not represent an in-principle ethical objection to its use. This is because, while ethical debates remain, scientific and technological shortcomings may be resolved. Therefore, as The Hinxton Group (2008) recommend, it is important that these concerns are distinguished. In doing so, one may consider the ethical objections that could be posed even against the technology in the safest form that could reasonably be expected. If our objections are solely based upon safety concerns – as are those of the HFEA – then we are unprepared in the event of their future circumvention. Moreover, uncertainties are inherent in all medical interventions; an in-principle objection on these grounds would also call into question treatments that have been approved and are now widely accepted such as* in vitro* fertilisation (IVF) and the mitochondrial replacement therapies. The benchmark for IVG approval should be based upon that of comparable technologies such as these.

## Ethical objections to reproductive *in vitro* gametogenesis

A systematic literature search was conducted to find the ethical objections regarding reproductive IVG. While this overview does not catalogue every objection posed, it attempts to present the most convincing arguments against allowing* in vitro*-derived gametes to be used for reproduction.

### New kinds of parenthood

IVG affords biological parenthood to more family constructions than does natural conception. Concerns regarding this fact constitute a large proportion of those found in the literature. Biological parenthood could conceivably be made accessible to the deceased; postmenopausal women; single individuals; same-sex couples; groups of more than two individuals; children, fetuses and embryos (Palacios-González *et al.* 2014).

Objections to the use of IVG in these circumstances arise more frequently than to opposite-sex reproduction because they can be seen to be non-therapeutic – they serve a social rather than a medical goal and are not seen to align with the purpose of medical practice (Notini *et al.* 2020). It is generally agreed that medicine serves to improve the health of the population; as a result, determining which interventions are therapeutic comes down to our conception of health and disease of which there are two competing theories. Naturalism defines disease as a deviation from natural functioning and so sympathises with this view that opposite-sex IVG is fundamentally different. However, normativism sees disease as a state in which one would rather not be. In this spirit, the World Health Organization (WHO) (WHO 2020) defines health as ‘a state of complete physical, mental and social well-being and not merely the absence of disease or infirmity’. With normativism and the WHO in mind, it seems that both infertile opposite-sex couples and other kinds of prospective parents are considered diseased or are, at least, less healthy than fertile couples. Therefore, IVG could be seen as therapeutic in both scenarios.

However, it is not widely accepted that any ARTs are therapeutic in nature. In reality, the process of IVG does not restore normal biological functioning rather it allows the creation of offspring where there would not have been, independent of the parents’ health status. This argument also dispels the distinction between the use of IVG for opposite-sex couples and that for other kinds of prospective parents. Therefore, regardless of whether one views IVG as therapeutic or non-therapeutic for opposite-sex couples, the same conclusion should be drawn for other kinds of prospective parents.

To summarise, there is a strong ethical argument to suggest that the therapeutic status of reproductive IVG is equivalent for many kinds of prospective parenthood situations. Nevertheless, there may be valid reasons to prohibit IVG in some of these cases but not others. For example, it has been speculated that IVG in same-sex couples would necessitate genetic engineering of one of the induced pluripotent stem cells used. This is because it is likely to be difficult or impossible to derive eggs from chromosomally male cells and sperm from chromosomally female cells (The Hinxton Group 2008). Allowing the birth of genetically engineered offspring is a contentious topic but it is widely deemed unethical. In addition, safety concerns exist for solo parenthood in that the process is analogous to consanguineous reproduction and would likely lead to higher rates of recessively inherited genetic disorders.

In conclusion, one may construct a coherent argument for the approval of IVG in only a select few of these parenthood scenarios. At the very least, the mere possibility of the more ethically suspect parenthoods does not provide a justifiable ethical objection to its use in opposite-sex couples. It is clear that each of these situations should be considered separately and their unique risks and benefits analysed. Although there is a coherent basis for approving opposite-sex reproduction but not others, it must be said that these are not in-principle objections. If any one of these scenarios were found to be ethically equivalent to opposite-sex reproduction and incur no further safety concerns, their approval should, too, be permitted.

### Embryo farming, selective breeding and designer babies

IVG is seen by some as a gateway into what has been called ‘embryo farming’. This is the process whereby embryos are created on a large-scale, giving rise to their commodification. As the limitations of gamete and embryo donation are lifted by the use of* in vitro*-derived gametes, experimental fertilisation and embryo destruction could be performed at a much higher rate. Many see this as an affront to the moral status of the embryo and believe that embryos ought not to be treated like any other raw material.

Further to this, bioethicists fear that when combined with genetic modification techniques and pre-implantation genetic testing (PGT), embryo farming could facilitate selective breeding. In other words, this would allow prospective parents to choose their future offspring based upon the characteristics that they are predicted to exhibit.

Selecting for non-disease-related traits is particularly controversial – Segers *et al.* (2019*b*) say that this crosses the line between harm prevention and eugenics. There are also concerns that such practices will exacerbate inequality and discrimination and reduce population diversity (Glenn Cohen & Pearlman 2019). It may be seen as a means of creating an imbalance in the value of lives and some bioethicists have called it a manifestation of ‘an untoward desire for human perfectionism’. Segers (2017) stated that selective breeding failed to provide offspring with an ‘open future’, something to which they have a right.

Though selective breeding may be controversial, it must be said that IVG is neither necessary nor sufficient for selective breeding or embryo farming and so this argument should not undermine the acceptability of IVG overall (Segers *et al.* 2019*b*). As with PGT and IVF, regulation rather than prohibition of IVG is required.

### The slippery slope argument

Some have claimed that approving reproductive IVG is a gateway towards the approval of other, more controversial, treatments. They claim that the reasoning behind supporting IVG could be iterated thereby allowing the approval of further practices that society traditionally deems to be unethical. This situation is known as a ‘slippery slope’.

Segers *et al.* (2019*a*) present such an argument in their paper in which they argue that there is a strong ethical link between reproductive IVG and reproductive cloning. For this reason, they conclude that the verdicts on IVG and cloning approval should be the same. On this basis, one may indeed conclude that the approval of reproductive cloning should follow that of reproductive IVG. As a result, arguing for the latter may be seen as a slippery slope. However, if there are no coherent and justifiable ethical objections to reproductive cloning, then its approval ought to be enacted regardless of IVG approval. To argue that reproductive IVG approval is a slippery slope implies that the slope leads to unethical conclusions. In this case, the conclusion is not unethical and, as such, the approval of reproductive IVG is ethically sustainable – policymakers are not obliged to approve unethical technologies as a result of IVG approval.

### Interference with natural reproduction

Finally, one must explore the objections to reproductive IVG on the basis that it is unnatural and, as such, evokes instinctive suspicion, fear or disgust – the so-called ‘yuck factor‘. Of all the objections discussed, this will perhaps be the one most commonly held by the general public.

The importance of nature and naturalness to society is acknowledged in an analysis by the Nuffield Council on Bioethics (2015). From this, it is clear that the thoughts that underlie society’s reasons for caring about naturalness are diverse and complex. However, despite the cognizant rationale behind some of these ideas, they are difficult to defend from an ethical standpoint.

Ethicists discourage objections based on natural law as they have been illustrated to be flawed and morally prejudiced (Bredenoord & Hyun 2017). Even if this were not the case, an attack on the unnatural is a *prima facie* move which targets the entire medical profession, including medicines, vaccines and other ARTs. This is something that, one must assume, is not the intention of proponents of such a view.

Therefore, one may say instead that reproductive IVG somehow crosses a line and is more unnatural than other medical interventions but even this is difficult to justify. When one is less accustomed to a certain practice, it may attract more distrust or criticism than is warranted; this is a manifestation of the mere-exposure effect, a cognitive bias that renders individuals more averse to the unfamiliar. Such a belief does not reflect the moral value of the practice in question (Segers *et al.* 2019*a*). As an example, IVF was initially regarded as morally suspect for many years – it is only as its practice has become commonplace that public opinion has shifted in its favour. Therefore, while ethical policy should recognise pluralism, it should be developed from rational arguments that are accepted as valid from the perspective of all stakeholders (Hendriks *et al.* 2015).

Although the ethical basis for society’s attachment to naturalness is shaky and intuition is not always a reliable source of argument, one cannot dismiss these concerns. The scientific community must engage in discourse with those who hold oppositional stances in order to understand the reasoning behind their objections and education must be at the forefront of this effort.

## Conclusion

In light of the arguments presented in this review, I conclude that there is no coherent and justifiable in-principle ethical objection to the use of IVG as an ART for those who cannot, by any other means, parent offspring with whom they share genetic material. Although both practical and safety concerns currently prevent its application in humans, the approval of reproductive IVG ought to be enacted upon their resolution.

Though there is no coherent and justifiable ethical objection, it is wise not to dismiss public reluctance to accept new technologies. The successful implementation of reproductive IVG is contingent not only upon its ethical basis but also upon public opinion. For this reason, I suggest that the HFEA ought to soon start a public consultation on the ethical advisability of sanctioning the therapeutic use of IVG.

## Declaration of interest

The author declares that there is no conflict of interest that could be perceived as prejudicing the impartiality of this review.

## Funding

This work did not receive any specific grant from any funding agency in the public, commercial, or not-for-profit sector.
